# Decision support system for the operating room rescheduling problem

**DOI:** 10.1007/s10729-012-9202-2

**Published:** 2012-06-13

**Authors:** J. Theresia van Essen, Johann L. Hurink, Woutske Hartholt, Bernd J. van den Akker

**Affiliations:** 1 Center of Healthcare Operations Improvement and Research (CHOIR), University of Twente, Enschede, P.O.Box 217, 7500 AE Enschede, The Netherlands; 2 Isala Clinics, P.O.Box 10500, 8000 GM Zwolle, The Netherlands

**Keywords:** Operating rooms, Rescheduling, Integer programming, Decision support system

## Abstract

Due to surgery duration variability and arrivals of emergency surgeries, the planned Operating Room (OR) schedule is disrupted throughout the day which may lead to a change in the start time of the elective surgeries. These changes may result in undesirable situations for patients, wards or other involved departments, and therefore, the OR schedule has to be adjusted. In this paper, we develop a decision support system (DSS) which assists the OR manager in this decision by providing the three best adjusted OR schedules. The system considers the preferences of all involved stakeholders and only evaluates the OR schedules that satisfy the imposed resource constraints. The decision rules used for this system are based on a thorough analysis of the OR rescheduling problem. We model this problem as an Integer Linear Program (ILP) which objective is to minimize the deviation from the preferences of the considered stakeholders. By applying this ILP to instances from practice, we determined that the given preferences mainly lead to (i) shifting a surgery and (ii) scheduling a break between two surgeries. By using these changes in the DSS, the performed simulation study shows that less surgeries are canceled and patients and wards are more satisfied, but also that the perceived workload of several departments increases to compensate this. The system can also be used to judge the acceptability of a proposed initial OR schedule.

## Introduction

The Operating Room (OR) department is one of the most expensive resources of a hospital. However, managing the OR department is hard due to conflicting priorities and preferences of stakeholders. Therefore, planning and scheduling methods are needed to increase the efficiency in OR departments. See Cardoen et al. [3] and Hulshof et al. [12] for an overview on OR planning and scheduling.

In this paper, we focus on the rescheduling of surgeries, or more precisely, on the rescheduling of surgeries throughout the day. On the one hand, emergency patients who need surgery arrive throughout the day. In many hospitals, these surgeries are scheduled in one of the elective ORs which disrupts the OR schedule. On the other hand, a change in the surgery duration of elective surgeries may also disrupt the OR schedule. Therefore, the initial OR schedule may have to be adjusted throughout the day to ensure that it is still possible to execute the schedule. The new OR schedule must fulfil quite a number of restrictions, and in addition, there are several stakeholders whose preferences and priorities must be met. Since it is hard for an OR manager to consider all these restrictions and preferences simultaneously, we develop a decision support system (DSS) which supports the OR manager with rescheduling the ORs. The approach proposed in this paper is general, but the realization and used preferences are based on data from a hospital in the Netherlands.

Most existing literature focuses on operational off-line scheduling, which is done one or several days before surgery, instead of operational on-line scheduling, which is done on the day of surgery. The papers concerning operational off-line scheduling mainly focus on two methods. The first method is reserving time for emergency surgeries to minimize overtime and maximize OR utilization (see e.g. [2, 10, 13]). The second method is sequencing the elective surgeries such that the overtime caused by surgeries with a longer duration than expected is minimized (see e.g. [4, 14, 16]). In addition, some papers concerning operational off-line scheduling include the preferences of stakeholders. For example, Marcon and Dexter [17] consider the preferences of the holding and recovery departments and Marjamaa et al. [18] consider the preferences of the anesthetists.

One of the papers concerning operational on-line scheduling is the paper by Dexter [5] who examined whether moving the last surgery of the day to another OR could decrease overtime labour costs. The developed statistical strategy was based on historical data. However, in practice, often one surgeon operates in an OR on a day or part of the day and therefore, it is not allowed to move a surgery to another OR.

Dexter et al. [8] introduce four ordered priorities on which an OR management decision for changing the OR schedule can be based. The first and most important priority is patient’s safety. The second priority states that a surgery can only be canceled if the patient safety is not endangered. The third priority is to minimize overtime, and the fourth and last priority is to reduce patient waiting times. These priorities, however, put minimizing overtime above the patients timing preferences. For patients it is not preferred to schedule their surgery earlier and certainly not later in the day. In addition, no priority considers the workload level on other departments like wards, the holding department, and the recovery department.

Another paper of Dexter et al. [7] considers the sequencing of urgent surgical cases. They proposed a sequencing which is based on the following three objectives: (i) minimize the average waiting time of surgeons and patients, (ii) sequence the surgeries in order of appearance, and (iii) schedule the surgeries in order of medical urgency. However, none of these objectives consider the preferences of the elective patients and other departments.

It seems that none of the existing papers on OR rescheduling considers the preferences and priorities of all the stakeholders simultaneously. This paper tries to fill this gap. Based on a given case in a Dutch hospital, we mainly focus on personal preferences and less on economical preferences, because patient and personnel satisfaction is highly important in the Netherlands due to shortage of personnel and competition between hospitals. In Section 2, we discuss the stakeholders and their restrictions and preferences which are based on a survey performed at the Isala Clinics, which is the above mentioned hospital in the Netherlands. Although these restrictions and preferences may differ between hospitals, the principle ideas of the method developed in this paper should be applicable for other hospitals too. Because we want to develop a DSS that generates adjusted OR schedules within a short amount of time, we first analyze the problem to determine which changes are preferred based on the preferences of the stakeholders. Therefore, we incorporate the restrictions and preferences in an Integer Linear Program (ILP), which has as goal to minimize the deviation from the preferences of the stakeholders. Although we prove that the problem is strongly NP-hard for two or more ORs, we are able to solve this ILP due to the moderate size of the instance. The solutions to the ILP are used to determine the changes made in the optimal OR schedule for instances of the Isala Clinics. These changes are incorporated as decision rules in the DSS which is described in Section 4. As we incorporate the preferences of all stakeholders and as these preferences are given by the stakeholders themselves, we do not expect that the resulting changes are subject to psychological bias as mentioned in other papers (see e.g. [6, 15]). The developed DSS is tested by means of a simulation study to determine what improvements can be made to the OR-schedule when the developed DSS is used in practice. The computational results of this simulation study are given in Section 5. Section 6 draws conclusions and gives recommendations for further research.

## Problem formulation

In this section, we give an introduction to the OR rescheduling problem and we introduce an ILP model which can be used to determine a new OR schedule throughout the day. The ILP includes all relevant constraints that are imposed on the OR schedule; e.g. the availability of a patient, as well as the availability of an OR with OR assistants, a surgeon and an anesthetist. In addition, the capacity of the holding and recovery department are considered. A detailed description of the constraints is given in the following subsections.

The objective of the ILP is to minimize the deviation of the preferences for the involved stakeholders. When the OR schedule deviates from these preferences some penalty costs are incurred and the weighted sum of these penalty costs is minimized. There can be, for example, penalty costs for deviating from the scheduled start time of a surgery or for the amount of resulting overtime. In addition, we minimize the number of canceled patients, as this is not preferred by any of the stakeholders. The developed ILP can also be used to determine whether a proposed OR schedule is feasible or not, and in case it is feasible, to calculate the deviation from the preferences of the stakeholders.

**Fig. 1:**
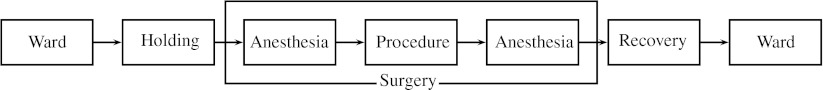
Patient process

Before we introduce the model, we first give a short description of the process a patient follows on the day of surgery in the Isala Clinics (see Fig. 1). On or before the day of surgery, the patient is admitted on a ward where he/she is prepared for surgery. Some time before surgery, the patient is transported to the holding department where the patient is further prepared for surgery. Then, the patient is transported to the operating room where the anesthetist administers anesthesia. After this, the surgeon performs the surgical procedure. When the surgical procedure is finished, the anesthetist reverses the anesthesia, and then, the patient is transported to the recovery department where he/she recovers from the effects of the anesthesia. At the time these effects have completely worn off and the patient’s condition is considered stable, he/she is transported back to the ward. Emergency patients are not first admitted on one of the wards, but are directly transported to the holding department or, in some very urgent cases, to the emergency room in which the surgery will take place. After surgery, these emergency patients follow the same path as the elective patients.

For the modeling, we discretize an OR-day into *T* time periods which have a length of *δ* minutes. The length of one OR-day is therefore *δT* minutes. We denote by time *t* ∈ *T* the period ((*t* − 1)*δ*, *tδ*]. The set of ORs is given by set *J* and consists of *M* ORs. The start time of OR *j* ∈ *J* is denoted by *S*_*j*_ and the end time by *F*_*j*_. The set of surgeries is given by set *I* and consists of *N* surgeries. The subset $I_j\subseteq I$ denotes the surgeries that are scheduled in OR *j* ∈ *J* and *O*_*i*_ ∈ *J* denotes the assigned OR for surgery *i* ∈ *I*.

The initial OR schedule, which is given at the beginning of the day and which has already been determined one or several days before, is defined by the assignment of the elective surgeries to an OR and the initially planned start times *P*_*i*_ of the elective surgeries. Each surgery has an expected duration *E*_*i*_ which includes the time for administering and reversing anesthesia, however, in practice, the duration of a surgery generally deviates from this duration and takes longer or shorter than expected. When a surgery takes less time than expected, and the next surgery starts at its assigned time *P*_*i*_, the initial OR schedule is not disrupted. However, it may be beneficial for the OR and other departments to schedule this next surgery earlier. When a surgery takes longer than expected, the next surgery may have to start later. This results in a shift of the not yet started surgeries in this OR. Because of this, some resource constraints may be violated. In addition to these deviations of the durations of the elective surgeries, emergency surgeries may arrive which also disrupt the initial OR schedule. Therefore, throughout the day, a new OR schedule may have to be created for all not started elective and emergency surgeries. In the following, we denote by set *I* the set of all these elective and emergency surgeries. The rescheduling is done by assigning a new start time to each surgery *i* ∈ *I*. Formally, this is expressed by binary variables *s*_*it*_, which are one when surgery *i* ∈ *I* starts at time *t* ∈ *T*, and zero otherwise. It is important to note that we do not allow the elective surgeries to be assigned to another OR, because each surgery has to be performed by the surgeon operating in the OR assigned to the surgery in the initial OR schedule. Thus, all elective surgeries have to be scheduled in the same OR as in the initial schedule. Because we only reschedule the not yet started surgeries, the start time of OR *j* ∈ *J* for the rescheduling problem is either given by the start time of the OR in the morning or the expected end time of the last started surgery in this OR.

Within the rescheduling, it may be necessary to cancel an elective surgery, for example because of an arriving emergency surgery. The decision variable *u*_*i*_ denotes whether elective surgery *i* ∈ *I* is canceled or not, i.e., the variable is one when the surgery is canceled and zero otherwise. When *u*_*i*_ is zero, the surgery is not canceled and therefore a new start time must be assigned, i.e., ∑ _*t* ∈ *T*_ *s*_*it*_ must be one in this case. When a surgery is canceled, the opposite holds, i.e., if *u*_*i*_ = 1 we must have ∑ _*t* ∈ *T*_ *s*_*it*_ = 0. This is ensured by the following constraint.

1 $$ \begin{array}{lll} \displaystyle\sum\limits_{t\in T} s_{it}=1-u_i,&{\kern24pt} & \forall i\in I \end{array} $$

Note that we do not consider the rescheduling of a canceled surgery, because we only focus on rescheduling within the day and not from day to day.

The new start time of surgery *i* ∈ *I* should fulfil a number of constraints. It should be greater than or equal to (i) the ready time of the patient which is given by *Y*_*i*_, (ii) the start time of the assigned surgeon *C*_*i*_ which is given by $D_{C_i}$, and (iii) the start time of the assigned OR *O*_*i*_. The following constraint ensures this.

2 $$ \begin{array}{lll} s_{it}=0,& {\kern24pt} & \forall i\in I, t<\max\left(S_{O_i},Y_i,D_{C_i}\right) \end{array} $$

The subset *I*_*MD*_ ⊂ *I* denotes the set of surgeries that should start before a certain time because of medical reasons. This medical deadline of surgery *i* ∈ *I* is given by *L*_*i*_. Furthermore, it is not allowed to cancel these surgeries, i.e., we must have *u*_*i*_ = 0 and the surgery has to start before *L*_*i*_.

3 $$ \begin{array}{lll} \displaystyle\sum\limits_{t=0}^{L_i} s_{it}=1,& {\kern24pt} &\forall i\in I_{MD}\\ u_i=0,&{\kern24pt} & \forall i\in I_{MD} \end{array} $$

The decision variable *s*_*it*_ and *u*_*i*_ completely determine the new OR schedule. However, to model the other restrictions and preferences some extra variables have to be defined which are introduced at the places where they are needed.

**Fig. 2:**
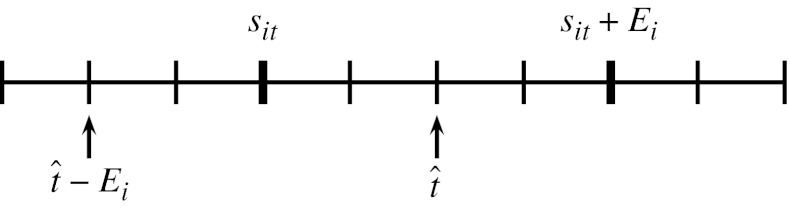
Determine *b*_*it*_

In each OR, only one surgery can be performed at a time. To model this, we need to determine if a surgery is ongoing at time *t* ∈ *T*. For this, we introduce the binary variables *b*_*it*_ which are one when surgery *i* ∈ *I* is performed on time *t* ∈ *T* and zero otherwise. A surgery is ongoing on time *t* ∈ *T* when the start time of surgery *i* ∈ *I* is between time *t* and time *t* − *E*_*i*_. This is shown in Fig. 2 and expressed by the following constraint.

4 $$ b_{it}=\displaystyle\sum\limits_{\hat{t}=t-E_i+1}^{t} s_{i\hat{t}},\qquad \forall i\in I,t\in T $$

The following constraint ensures that for each OR only one surgery can be performed at a time.

5 $$ \displaystyle\sum\limits_{i\in I_j} b_{it}\leq 1, \qquad \forall j\in J,t\in T $$

The above constraints describe some of the hard constraints for the rescheduling process resulting from the situation in the OR. In the following subsections, we describe and model the involved stakeholders. For each stakeholder, we describe the tasks the stakeholder has to perform during the day, the restrictions they impose on the OR schedule, the impact a change in the OR schedule has on the stakeholder and the preferences of the stakeholder. The impacts are modeled by linear constraints, and penalty costs for deviating from the preferences are incorporated in the objective function. The chosen functions and corresponding parameters used to describe the preferences of the stakeholders and the penalty costs for deviating from these preferences are determined based on a survey at the Isala Clinics (see Appendix A). Applying the model to a different hospital asks for an analysis of the preferences of the stakeholders in this hospital and may lead to other penalty cost functions. However, the principle structure of the proposed approach does not have to be adapted.

### Patient

The key stakeholder is the patient. For patients it is important that the surgery takes place at the scheduled time. Penalty costs are incurred when the new start time deviates from this preference. In order to determine the total penalty costs, we need to know the new start time of the surgery in the OR schedule. This time is denoted by the variable *w*_*i*_, and in case surgery *i* ∈ *I* is canceled, we define *w*_*i*_ to be equal to the start time of surgery *i* ∈ *I* in the initial OR schedule.

6 $$ w_i=\displaystyle\sum\limits_t ts_{it}+u_iP_i, \qquad \forall i\in I $$

If we now denote by *y*_*i*_ the difference of the initial and new start time of surgery *i* ∈ *I*,

7 $$ y_i=w_i-P_i, \qquad \forall i\in I $$

this variable *y*_*i*_ is zero when surgery *i* ∈ *I* is canceled or when the start time of surgery *i* ∈ *I* has not changed. For emergency surgeries, we take as initial planned start time *P*_*i*_ the time the patient arrived at the hospital. This ensures that emergency surgeries are scheduled as soon as possible. The variable *y*_*i*_ is negative when surgery *i* ∈ *I* starts earlier in the new OR schedule and when *y*_*i*_ is positive, surgery *i* ∈ *I* starts later. As patients judge earliness and tardiness different, we split the variable *y*_*i*_ in two cases by introducing variables $y^{\rm later}_i$ and $y^{\rm earlier}_i$. The variable $y^{\rm later}_i$ takes value *y*_*i*_ when *y*_*i*_ is positive, and variable $y^{\rm earlier}_i$ takes value − *y*_*i*_ when *y*_*i*_ is negative, which is ensured by the following constraints and the fact that the objective tries to minimize these variables.

8 $$ \begin{array}{lll} y^{\rm earlier}_i\geq P_i-w_i,& {\kern24pt} & \forall i\in I\\ y^{\rm earlier}_i\geq 0, & {\kern24pt} &\forall i\in I\\ y^{\rm later}_i\geq w_i-P_i,& {\kern24pt}& \forall i\in I\\ y^{\rm later}_i\geq 0, & {\kern24pt}& \forall i\in I\\ \end{array} $$

Note that constraints (8) replace constraint (7) in the ILP.

Based on the survey in the hospital, we concluded that patients assign different penalty costs to different values of *y*_*i*_. To model this, a function *f*_*PT*_(*y*_*i*_), denoting the penalty costs when surgery *i* ∈ *I* is shifted *y*_*i*_ time periods, is introduced. This function is also split into two parts, namely $f^{\rm earlier}_{PT}(y^{\rm earlier}_i)$ and $f^{\rm later}_{PT}(y^{\rm later}_i)$.

**Fig. 3:**
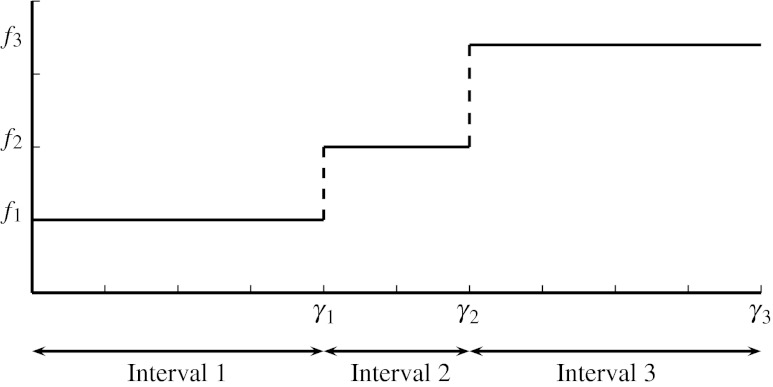
Step function

Based on the patient survey, the penalty cost functions can be modeled best by step functions which are combinations of linear functions, see Fig. 3. The specific value of the steps are also given by the questionnaire. To determine the correct value of the function $f^{\rm earlier}_{PT}$ for a specific value of $y^{\rm earlier}_i$, we introduce two parameters. The first is *f*_*k*_, which denotes the function value in interval *k*, and the second is *γ*_*k*_, which denotes the right endpoint of interval *k*.

To be able to incorporate these step functions in an ILP model, we introduce binary variables *λ*_*ik*_ which are one when $y_i^{\rm earlier}$ is in interval *k* and zero otherwise. This is ensured by the following two constraints.

9 $$ \begin{array}{lll} \displaystyle\sum\limits_k \lambda_{ik}\gamma_k\geq y^{\rm earlier}_i,& {\kern24pt} & \forall i\in I \\ \displaystyle\sum_k \lambda_{ik}=1, & {\kern24pt} &\forall i\in I \end{array} $$

The value of the penalty function is now determined by:

10 $$ \begin{array}{lll} f_{PT}^{\rm earlier}\left(y_i^{\rm earlier}\right)=\displaystyle\sum\limits_k \lambda_{ik}f_k,& {\kern24pt} &\forall i\in I. \end{array} $$

The total penalty costs of the patient group is given by *p*_*PT*_, and is defined as the sum of the penalty costs of each patient.

11 $$ p_{PT}=\sum\limits_{i\in I} \left(f^{\rm earlier}_{PT}\left(y^{\rm earlier}_i\right)+f^{\rm later}_{PT}\left(y^{\rm later}_i\right)\right) $$

Note that the two penalty functions $f^{\rm earlier}_{PT}$ and $f^{\rm later}_{PT}$ in general have different parameters. Because we minimize the total penalty costs, this method is only applicable for non-decreasing step functions.

### Ward

Prior to surgery, the patient is admitted to a ward. On this ward, the patient is prepared for surgery. The survey showed that when a surgery starts earlier than scheduled, the workload on the ward increases if the patient is not ready yet. When a surgery starts later than scheduled, the workload can also increase. Therefore, penalty costs are incurred when there is a change in the start time of a surgery. The total penalty costs are calculated in the same way as for the patient. Based on the outcome of the survey a step function *f*_*W*_(*y*_*i*_) is defined, which denotes the penalty costs for the wards if the start time of a surgery is shifted for *y*_*i*_ time periods. Note, that we do not distinguish between a surgery being scheduled earlier or later. The total penalty costs for wards is then given by *p*_*W*_ = ∑ _*i* ∈ *I*_ *f*_*W*_(*y*_*i*_).

### Holding department

After the preparation on the ward, the patient is transported to the holding department where he/she is prepared further. The length of stay of patients on the holding department is given by *V* which can be longer than the preparation time needed. The holding department has a limited number of beds *O*_1_ which provides a maximum for the number of patients treated at this department at the same time. Another limit on the number of patients who can be treated simultaneously is given by the available number of nurses at time *t* ∈ *T* which is denoted by *X*_*t*_. A nurse needs *ρ* minutes to prepare a patient, implying that *X*_*t*_ nurses can prepare at most $\frac{\delta}{\rho}X_t$ patients in time period *t*. Concluding, we define the capacity of the holding at time *t* by $\min\left(O_1,\frac{\delta}{\rho}X_t\right)$. The number of patients present on the holding on time *t* ∈ *T* is denoted by *l*_*t*_ and is given by:

12 $$ \begin{array}{lll} l_t=\sum_{i\in I}\sum_{\hat{t}=t+1}^{t+V}s_{i\hat{t}},&{\kern24pt} &\forall t\in T \end{array} $$

This number should be smaller than or equal to the capacity of the holding which is ensured by the following constraint.

13 $$ \begin{array}{lll} l_t\leq \min\left(O_1,\frac{\delta}{\rho}X_t\right),&{\kern24pt} & \forall t\in T \end{array} $$

**Fig. 4:**
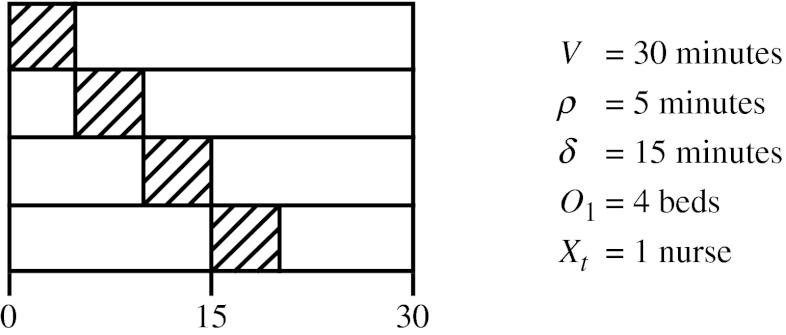
Excluded feasible solution

Note that when $\frac{\delta}{\rho}X_t\leq O_1$ for some *t* ∈ *T*, constraint (13) may exclude some feasible solutions (for an example, see Fig. 4). If we want to prohibit this, we also need to schedule the preparation time of the patients. However, this increases the complexity of our problem. Note that this issue does not occur when the length of stay *V* equals *δ* which is the case for the instances used.

The survey at the Isala Clinics showed that the holding department prefers a levelled amount of patients that are present at each point of time. Therefore, penalty costs given by the step function *f*_*HD*_(*l*_*t*_), are incurred when the number of patients *l*_*t*_ exceeds a certain threshold. The penalty costs for different values of *l*_*t*_ are specified by the manager of the holding department. As at the beginning of the day (until a prespecified time *ψ*), personnel from the recovery department assist on the holding department (no patients are present at the recovery department at this time), penalty costs are only incurred from time *ψ* on. The total penalty costs *p*_*HD*_ are given by $\sum_{t=\psi+1}^{T}f_{HD}(l_t)$.

### Anesthetist

The anesthetist is responsible for administering and reversing anesthesia on one or more ORs. Therefore, he/she has to administer and reverse all anesthesias in these ORs. However, during the surgical procedure, the anesthetist does not have to be present in the OR, because the presence of an anesthesia nurse is enough. Therefore, similar to constraints (4) and (5), we include constraints which prohibits that more than one anesthesia is administered or reversed at a time in the ORs to which the anesthetist is assigned. For more details, see [11].

However, there are a few exceptions. When a surgery is complex, for example when the patient is younger than 6 months, the anesthetist must be present during the complete surgery which includes the surgical procedure. This means that during this time no anesthesia can be administered or reversed in one of the other assigned ORs. This is also ensured by constraints similar to constraint (5).

### Surgeon

The surgeon is assigned to one OR and only has to perform the surgical procedure. This means that he/she does not have to be present during administering and reversing anesthesia. The constraints that ensure this are of the same structure as constraints (4) and (5).

### OR assistants

The OR assistants do not impose any restrictions on the OR schedule. Their only preference is that overtime is minimized. Overtime can occur because of arriving emergency surgeries and surgeries whose duration is longer than expected. Therefore, penalty costs are incurred when there is overtime. The amount of overtime in OR *j* ∈ *J* is denoted by variable *o*_*j*_. The value of this variable *o*_*j*_ is calculated by the following constraint.

14 $$ \begin{array}{lll} o_j=\displaystyle\sum\limits_{i\in I_j}\displaystyle\sum_{t=F_j+1}^T b_{it}, & {\kern24pt} & \forall j\in J \end{array} $$

The step function *f*_*OS*_(*o*_*j*_) provides the penalty costs for OR *j* ∈ *J* when overtime of *o*_*j*_ time periods is incurred. The OR assistants specified the penalty costs for different values of *o*_*j*_. The total penalty costs for the OR assistants is given by *p*_*OS*_ = ∑ _*j* ∈ *J*_ *f*_*OS*_(*o*_*j*_).

### Recovery department

After surgery, the patient is transported to the recovery department. Here, the patient is monitored while he/she recovers from surgery. The length of stay on this department varies with the expected duration of the surgery and is given by $\max(U, \frac{1}{2} E_i)$, where *U* is the minimum length of stay on this department. The number of patients present at the recovery department at time *t* ∈ *T* is denoted by *z*_*t*_ and can be determined in the same way as for the holding department. The capacity of the recovery department is restricted by the number of beds *O*_2_.

Another restriction is given by the number of patients who can be treated simultaneously, which depends on the number of available nurses *R*_*t*_ at time *t* ∈ *T*. Each nurse can monitor *φ* patients at a time, and therefore, *φR*_*t*_ patients can be treated simultaneously. Combining this with the number of beds *O*_2_, the capacity of the recovery at time *t* ∈ *T* is given by $\min\left(O_2,\varphi R_t\right)$. The number of patients present on the recovery department at time *t* ∈ *T* should be less than or equal to this capacity. This is ensured by constraints that are of the same structure as constraints (12) and (13).

Like the holding department, the recovery department also prefers a levelled amount of patients that are present at each point of time. Therefore, penalty costs are incurred when the number of patients exceeds a certain threshold. This is modeled by the step function *f*_*RC*_(*z*_*t*_) which provides the penalty costs incurred when *z*_*t*_ patients are present at time *t* ∈ *T*. The total penalty costs for the recovery is then given by *p*_*RC*_ = ∑ _*t* ∈ *T*_ *f*_*RC*_(*z*_*t*_).

### Radiology department

For some surgeries, an X-ray machine is needed during surgery. These surgeries are given by the set $I_{RL}\subseteq I$. For these surgeries a radiology technician should be present during administering anesthesia and the surgical procedure. This means that he/she does not have to be present during reversing anesthesia. The set of radiology technicians is given by set *K* and consists of *χ* radiology technicians. We restrict the number of required radiology technicians *d*_*t*_ at time *t* ∈ *T* to be smaller than or equal to the number of available radiology technicians. The constraints that ensure this are similar to constraints (4) and (5).

The survey showed that it is important for the radiology department that their employees at the OR department finish as early as possible such that they can carry out other work at the radiology department. Therefore, penalty costs are incurred when a radiology technician finishes later than needed, i.e., when the time the radiology technicians are present is longer than the time the radiology technicians are needed. In the following, we show how this is incorporated in the ILP.

For all radiology technicians *k*, we determine when their work is finished at the OR. These finish times are denoted by *τ*_*k*_ and are calculated by determining the latest time period where at least *k* radiology technicians where needed. First, for each time period *t* ∈ *T*, we introduce binary variables $\tilde{d}_{tk}$ which are one when *k* or more radiology technicians are needed in time period *t*, and zero otherwise. This is ensured by the following constraint.

15 $$ \begin{array}{lll} \tilde{d}_{tk}\geq \frac{d_t-k+1}{\chi},& {\kern24pt} &\forall t\in T, k\in K \end{array} $$

The finish time *τ*_*k*_ of radiology technician *k* is now given by the latest time period that $\tilde{d}_{tk}$ is equal to one, i.e.,

16 $$ \begin{array}{lll} \tau_k\geq t\tilde{d}_{tk},& {\kern24pt} & \forall t\in T, k\in K. \end{array} $$

Using the above constraints and the fact that we minimize the working time of the radiology technicians, *τ*_*k*_ denotes the time the radiology technicians finish. However, these values do not equal the number of time periods they are actually present at the OR. To obtain this value, the start time and break time should be subtracted. The start time of the radiology technicians is given by min _*j*_ *S*_*j*_, i.e., the start time of the OR department. For other hospitals, the start times of the radiology technicians may not be fixed. When this is the case, the start times can be determined with constraints similar to (15) and (16). In addition, all radiology technicians have a break of 45 min, i.e., $\frac{45}{\delta}$ time periods. The amount of periods the radiology technicians are having a break is thus given by $\upsilon=\frac{45\chi}{\delta}$. Therefore, the amount of time periods the radiology technicians are present at the OR is given by ∑ _*k*_ *τ*_*k*_ − *χS*_*j*_ − *υ*. This is an underestimation in case one or more radiology technicians finish before their break. However, we expect that this will rarely happen in practice.

The amount of time periods the radiology technicians are actually working at the OR is given by $\sum_{i\in I_{RL}} (E_i-Q_2)$, where *Q*_2_ is the amount of time it takes to reverse anesthesia and *E*_*i*_ is the duration of surgery *i* ∈ *I*_*RL*_. Now, the variable *x* defined by

17 $$ x =100\left(\frac{\sum_k \tau_k-\chi S_j-\upsilon }{\sum_{i\in I_{RL}}(E_i-Q_2)+1}\right) $$

denotes the inverse of the fraction of time the radiology technicians are busy. The step function *f*_*RL*_(*x*) denotes the penalty costs incurred for a value of *x*, specified by the radiology department, and gives the total penalty costs *p*_*RL*_ incurred.

### Pathology department

During some surgeries, tissue is removed from a patient which needs to be examined by a pathologist. These surgeries are denoted by the set $I_{PA}\subseteq I$. After the surgical procedure, the tissue is transported from the OR to the pathology department. When tissue arrives after closing time, overtime is incurred. Let *q*_*i*_ be an integer variable which denotes the amount of overtime which would be created by a single surgery *i* ∈ *I*, i.e., the number of time periods the tissue arrives late plus the examination duration *W*.

As after closing time only one pathologist is available at the pathology department, the available pathologist has to successively process the tissues that arrive late. In most cases this means that the pathologists has to work $\displaystyle\sum\limits_{i\in I,q_i>0} W$ periods in overtime. However, sometimes tissue will arrive so late, that the amount of overtime equals max _*i* ∈ *I*_ *q*_*i*_. Therefore, the amount of overtime *q*_total_ is estimated as follows:

18 $$ q_{\rm total}=\max \left(\max\limits_{i\in I} q_i,\sum\limits_{i\in I, q_i>0}W\right) $$

When two sets of tissue arrive really late at approximately the same time, this number is a lowerbound on the amount of overtime. However, this situation is not likely to occur in practice.

Again, a step function *f*_*PA*_(*q*_total_) is used to express the penalty costs incurred for a value of *q*_total_ and represents the total penalty costs *p*_*PA*_ incurred for the pathology department.

### Logistic department

The logistic department is responsible for preparing materials needed during surgery. The materials are laid out in the order in which the surgeries are scheduled. When two surgeries are interchanged, the logistic assistant incurs penalty costs, because they have to change the order in which the materials are laid out. Two surgeries *i* ∈ *I* and $\hat{i}\in I$ can only be interchanged when they are scheduled in the same OR, i.e., when $O_i=O_{\hat{i}}$. These two surgeries are interchanged when $(P_i-P_{\hat{i}})(w_{\hat{i}}-w_i)>0$, where *P*_*i*_ is the start time in the initial OR schedule and *w*_*i*_ is the start time in the new OR schedule. When this holds, we either have that both $(P_i-P_{\hat{i}})$ and $(w_{\hat{i}}-w_i)$ are positive or that both are negative. When both are positive, we have that $P_i>P_{\hat{i}}$ and $w_i<w_{\hat{i}}$. This means that in the initial OR schedule, surgery *i* ∈ *I* was scheduled later than surgery $\hat{i}\in I$ and that in the new OR schedule, surgery *i* ∈ *I* was scheduled earlier than surgery $\hat{i}\in I$. When both are negative, we have the opposite case.

We introduce binary variables $\kappa_{i\hat{i}}$ which are one when surgery *i* ∈ *I* and $\hat{i}\in I$ are interchanged and zero otherwise. This is ensured by constraints (19), where *T* is the number of time periods per day. When $(P_i-P_{\hat{i}})(w_{\hat{i}}-w_i)>0$, the variable $\kappa_{i\hat{i}}$ is set to one, however, when $(P_i-P_{\hat{i}})(w_{\hat{i}}-w_i)\leq 0$ the variable $\kappa_{i\hat{i}}$ could be set to either one or zero. But because we want to minimize the number of exchanged surgeries, the variable $\kappa_{i\hat{i}}$ gets the value zero.

19 $$ \left(P_i-P_ {\hat{i}}\right)\left(w_{\hat{i}}-w_i\right)\leq T^2\kappa_{i\hat{i}}, \forall \left(i>\hat{i}\right)\in I, O_i=O_{\hat{i}} $$

Let *f*_*LD*_ be the penalty cost incurred when two surgeries are interchanged, which value is specified by the logistic department. Then the total amount of penalty cost *p*_*LD*_ incurred for the logistic department is given by

20 $$ p_{LD}=\sum\limits_{j\in J}\sum\limits_{(i,\hat{i})\in I_j, i>\hat{i}}\kappa_{i\hat{i}}f_{LD}. $$

### Objective function

The goal of our model is to minimize the deviation from the preferences of the stakeholders. We denote the set of stakeholders by *Π* and we consider each stakeholder to be equally important. Since the order of magnitude of the cost functions introduced for the different stakeholders may differ, we have to introduce a weighted sum of the penalty costs *p*_*π*_ to compensate these differences. In this function, the priority *β*_*π*_ assigned to stakeholder *π* ∈ Π is determined such that all stakeholders contribute approximately the same amount to the objective function value. The general concept of the weighted sum of penalties has furthermore the advantage that by varying the priorities, we can develop, for example, also a more patient centred OR schedule.

Next to the penalty costs for deviation from the preferences of stakeholders, we also include penalty costs *η* for canceling a surgery. These penalty costs are set such that they contribute more than the combined total penalty costs of the stakeholders in case surgeries are canceled. This way, it is clear that canceling a surgery is not preferred, however, if needed it is possible to do it. Note that the resulting changes in the OR schedule are not risk-averse (see [19]), because by given the objective ‘minimizing the number of cancellations’ a very high weight, the OR utilization is maximized. Summarizing, the objective function is given by

21 $$ \min \sum\limits_{\pi\in \Pi}\beta_{\pi}p_{\pi}+\sum\limits_{i\in I} \eta u_i. $$

### Problem complexity

The problem introduced in the previous subsections has been modeled as an ILP. The following theorem justifies this approach, since it shows that efficient exact approaches are unlikely to exist.

#### **Theorem 1**

*The OR rescheduling problem is strongly NP-hard for two or more operating rooms.*

#### Proof

We prove the theorem by reducing 3-partition to the OR rescheduling problem. The 3-partition problem can be formulated as follows. Given positive integers *a*_1_,...,*a*_3*t*_, and *b* with $\sum_{j=1}^{3t} a_j=tb$, do there exist *t* pairwise disjoint subsets $R_k\subset \lbrace 1,\ldots,3t\rbrace$ such that $\sum_{j\in R_k}a_j=b$ for *k* = 1,...,*t*? The 3-partition problem is proven to be strongly NP-hard (see Garey and Johnson [9]).

The reduction is based on the following transformation, where we set the priorities for the patient, ward, and the holding, recovery, radiology, pathology and logistic department to zero. Therefore, we only aim to minimize overtime and the number of cancellations. Furthermore, we consider 2 ORs which have their own anesthetist and 6*t* − 2 surgeries with the following processing times and ready times:

$$ \begin{array}{lll} E_i=b,&Y_i=0&\forall 1\leq i\leq t-1, \\ & & i\in I_1, I_{RL},\\ E_i=a_{i-t+1},&Y_i=0&\forall t\leq i\leq 4t-1, \\ && i\in I_1,\\ E_i=b,&Y_i=2b(i-4t)&\forall 4t\leq i\leq 5t-1, \\ & & i\in I_2, I_{RL},\\ E_i=b,&Y_i=b(i-(5t-1))&\forall 5t\leq i\leq 6t-2,\\ && i\in I_2. \end{array} $$

The end and start times of the 2 ORs are:

$$S_j=0, \quad F_j=(2t-1)b \; \mbox{for }\; j\in J. $$

The capacities of the holding and recovery departments are assumed to be larger than 6*t* − 2, thus we do not have to consider the given constraints for these departments. Furthermore, only one radiology technician is available, and therefore, we have to consider the given constraints for the radiology department. Our goal is to create an OR schedule with objective value less than or equal to zero.

**Fig. 5:**
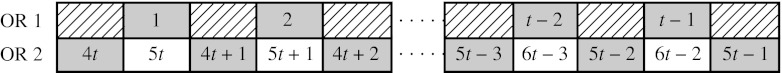
Reduction of 3-partition problem to the OR rescheduling problem

First note that, because of their ready times, the surgeries from $\lbrace 4t, 4t+1, \ldots, 6t-2\rbrace$ in OR 2 have to be scheduled as in Fig. 5 to achieve an objective value of zero, i.e., no overtime and cancellations may occur. In Fig. 5, the grey blocks denote surgeries that need a radiology technician and the white blocks denote surgeries that do not need a radiology technician. Because the surgeries from $\lbrace 1, 2, \ldots t-1\rbrace$ in OR 1 need a radiology technician, they have to be scheduled in the time intervals where the radiology technician is not busy in OR 2, i.e., as in Fig. 5. This leaves us with *t* blocks of length *b* in OR 1 which have to be filled with the surgeries from $\lbrace t, t+1, \ldots, 4t-1\rbrace$ to achieve zero overtime with zero cancellations, and thus, an objective value of zero. Therefore, our problem has a solution with objective value zero if and only if there exists a solution to the 3-partition problem. □

The proof of this theorem shows that already a very restricted version of the OR rescheduling problem is strongly NP-hard.

## Computational results ILP

We tested our ILP on data from the Isala Clinics, a hospital in the Netherlands. The data consists of 1168 surgeries scheduled over 27 days. The surgeries consist of 354 emergency surgeries, 193 surgeries who need X-ray, 79 surgeries during which tissue is removed, and 7 complex surgeries. The average expected duration of the surgeries is 103 min, and the average realized duration of the surgeries is 91 min. Because rescheduling is only performed during working hours, we removed the emergency surgeries that start before 07:30 and after 18:00. We implemented our model in AIMMS 3.10 and solved it with CPLEX 12.1 on an AMD Ahtlon X2 Dual Core L310 1.2 GHz processor with 4 GB RAM.

In the first two subsections, we discuss the parameter settings for the ILP model and the achieved results which are used to derive the decision rules for the DSS. In the last subsection, we determine the penalty costs for the initial OR schedule used at the Isala Clinics and the OR schedule realized at the end of the day. In addition, we optimize both the initial and realized OR schedule to show what improvements potentially can be realized when the developed method is used.

### Parameter settings

In this subsection, we discuss the parameter settings for the time periods and the priorities for each stakeholder.

**Fig. 6:**
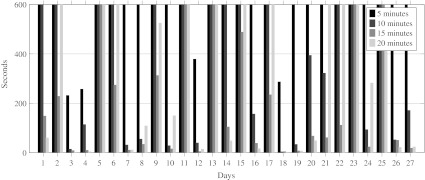
Runtime for different values of *δ*

To determine the appropriate length *δ* of the time periods, we solved the model for time periods of 5, 10, 15 and 20 min. We interrupted the ILP solver after 10 min of computation time. If after this time no optimal solution was found, we took the best solution found as our final solution. In Fig. 6, the runtime for each combination of day and *δ* is given.

**Fig. 7:**
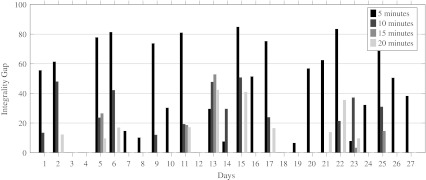
Integrality gap for different values of *δ*

We would expect that a smaller value of *δ* would increase the runtime of our model. For most days in our instance this holds, however, in some cases, the runtime for our model with *δ* equal to 15 min is shorter than the runtime for our model with *δ* equal to 20 min. Figure 7 shows that for *δ* equal to 5 min, an optimal solution was only found for 4 of the 27 days. For *δ* equal to 10 and 20 min, this number increased to 14 and 17 days, respectively. The model with *δ* equal to 15 min performs the best, because an optimal solution was found for 22 of the 27 days. This result seems to be the consequence of the input data, since most of the data is given in multiples of 15 min, for example, the expected surgery duration and the length of stay on the holding department.

**Fig. 8:**
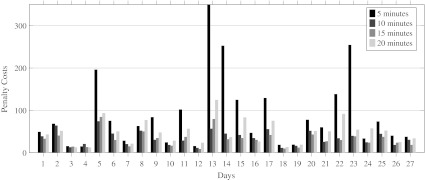
Objective function value for different values of *δ*

In Fig. 8, we give the objective function value for each combination of *δ* and day. If solved to optimality, the objective value should increase when *δ* increases, because there is more flexibility in the OR schedule when *δ* is lower, i.e., the model with *δ* = 5, should be able to provide the same or even a better solution than the model with *δ* set to 10, 15 or 20 min. However, Fig. 8 shows that the worst objective values are achieved when *δ* equals 5. This is because for most days no optimal solution was found within 10 min. From Fig. 8, we can conclude that our model with *δ* set to 15 min results in the lowest objective function value. Combining the results for the runtime and objective function, we choose to set *δ* to 15 min for further tests.

For each of the stakeholders, we have to determine its priority in the objective function. In the objective function, the total penalty costs of each stakeholder is multiplied by this priority. Our goal is that each stakeholder has approximately the same contribution in the objective function. In the following, we describe how we have determined the priority of each of the stakeholders.

First, we solve our model where all stakeholders have priority one. Next, we adjust the priorities in such a way that the weighted cost of each of the stakeholders for the achieved solution is approximately the same. This is done by setting the priority of the stakeholder with the lowest total penalty costs to one and the priorities of the other stakeholders such that their weighted costs equals the lowest total penalty costs. Table 1 shows the results if this method is applied to our data, where the total penalty costs are the average total penalty costs incurred per day.

**Table 1:** Total penalty costs and priorities

	Patient	Ward	Holding	OR assistants	Recovery	Radiology	Pathology	Logistics
Total penalty costs	7.45	5.26	0.00	1.11	1.91	1.00	0.56	0.91
Priority	0.08	0.11	1.00	0.50	0.29	0.56	1.00	0.62
Weighted costs	0.56	0.56	0.00	0.56	0.56	0.56	0.56	0.56

Note, that the holding department does not incur any penalty costs, because penalty costs are only incurred when four patients are treated simultaneously. The constraints imposed, however, limit the number of patients present on the holding department to three. This means that the holding department is not a bottleneck in the current situation.

We conducted some further tests where we varied these priorities slightly. The results from these tests show that patients, wards, and OR assistants have opposite interests compared to the recovery, radiology, pathology, and logistic department.

### Deriving decision rules

The main goal of applying the ILP model is to determine which adjustments to the initial OR schedule are allowed and preferred based on the given preferences of the stakeholders. To determine this, we use our ILP model to create at three points *t* ∈ *T* a new OR schedule which minimizes the deviation from the preferences of the stakeholders. For each of these three scenario’s, the initial OR schedule is given as input as well as the realization of the duration of the surgeries that started before time *t*. These realized durations may change the initial OR schedule, because this schedule was based on the expected durations. Since we cannot change the OR schedule for the already started surgeries, we start rescheduling at the new start time *S*_*j*_ of OR *j*, which we define as the end time of the last started surgery before time *t*. In addition, we schedule not yet started emergency surgeries that arrived before time *t*. We assign a new start time *s*_*it*_ to each elective and emergency surgery that has not started at time *t* or, when allowed, cancel this surgery such that the resource constraints are fulfilled and the deviation from the preferences is minimized. The three scenario’s are summarized below.

1. After 10 a.m.: In this scenario, the realized durations of the surgery that started before 10 a.m. are known. An emergency surgery is only included if it arrived before 10 a.m..
2. After 12 p.m.: In this scenario, the realized durations of the surgery that started before 12 p.m. are known. An emergency surgery is only included if it arrived before 12 p.m..
3. After 2 p.m.: In this scenario, the realized durations of the surgery that started before 2 p.m. are known. An emergency surgery is only included if it arrived before 2 p.m..

These three scenario’s are used to determine what adjustments our model makes to the OR schedule. For each of the scenario’s, we determine how often one of the following adjustments occurred: (i) shifting a surgery, (ii) exchanging the sequence of two surgeries, and (iii) canceling a surgery. In addition, we determine how often a break of a certain length was scheduled between two surgeries. The results are shown in Table 2.

**Table 2:** Results scenario 1, 2, and 3

	10 a.m.	12 p.m.	2 p.m.
Rescheduled surgeries	566	416	213
Shifted surgeries	375	297	176
Exchanged surgeries	1	0	0
Canceled surgeries	0	0	1
No break	264	183	71
Break 15 min	166	112	66
Break 30 min	62	33	21
Break 45 min	22	19	11
Break > 45 min	38	37	13
Mean break	15.84	18.71	19.78

Table 2 shows that shifting a surgery is the most frequent adjustment used, and often we see that a break is scheduled between two surgeries. The average length of a break is 15–20 min. When we only consider OR utilization, this may not seem to be optimal, however, these breaks can improve the perceived workload of other departments or may be necessary to fulfil the resource constraints. From the results we conclude that only two types of adjustments are preferred to be used. A surgery can be shifted or a break can be scheduled between two surgeries. This means that the order of surgeries stays the same during the day. So when we only allow these two adjustments, the number of feasible solutions decreases significantly, because we only have to consider one sequence of the surgeries instead of all possible sequences. Based on this, it is possible to develop a simple heuristic to determine a good OR schedule. A further benefit of this is that we do not need an expensive ILP solver to implement our approach.

Following the above considerations, we have incorporated this simple heuristic in a DSS which is described in Section 4.

### Potential improvements

To determine what improvements the DSS could potentially make compared to the OR schedules used at the Isala Clinics, we calculated, using the ILP model, the optimal initial OR schedule based on the expected surgery durations. Note, that for this optimization the assignment of the surgeries to an OR is given as in the given initial OR schedule. Thus, we only change the sequences of the surgeries in each OR. None of the surgeries can be canceled, and because it is an initial OR schedule, the change in the start time of the surgeries is not incorporated in the ILP model. This implies that the penalty costs for the patients, wards and logistic department are zero. Also, there are no emergency surgeries to be scheduled, because they have not arrived yet. This resulting fourth scenario is summarized below.

Scenario 4 Initial OR schedule: In this scenario, we compare the initial OR schedule used at the Isala Clinics with a new OR schedule determined by the ILP model, where we rescheduled all elective surgeries. Therefore, there are no emergency surgeries to be scheduled, and only the expected duration of each surgery is given.

In addition, we want to determine what improvements the DSS could make to the realized OR schedule. Filling in the actual surgery durations in the initial OR schedule will most likely result in an infeasible solution and because we do not know the exact decisions the OR manager will make, we can only guess what improvements can be achieved. To provide an upperbound on these improvements, and thus a lowerbound on the penalty costs, we optimized the realized OR schedule and compared it to the realized OR schedule provided by the Isala Clinics. The realized OR schedule of the Isala Clinics denotes how the surgeries were actually performed. This means that several changes have been made to the initial OR schedule, and therefore, we can determine the penalty costs achieved for these changes. For the optimal realized OR schedule, we assume that all realized durations of the elective and emergency surgeries are considered to be known in advance, and also the canceled surgeries are taken into account. In practice, this information is not known beforehand, and thus, this optimal realized OR schedule can not be achieved in practice. However, it provides a bound and, therefore, an indication of the room for improvement.

To define the input more precisely, we take all elective surgeries with their realized duration that were planned in the initial OR schedule, and in addition, we include all performed emergency surgeries with their realized duration. For the canceled surgeries, we use the given expected surgery durations. For this scenario, it can happen that surgeries are canceled or that their start time changes, which results in penalty costs for patients, ward, and the logistic department. This fifth scenario is summarized below.

Scenario 5 Realization: This scenario consists of all the elective surgeries scheduled in the initial OR schedule and all emergency surgeries that arrived during the day. The realized duration of all surgeries is known.

Table 3 provides the average total penalty costs per day for each of the stakeholders and compares the initial and realized OR schedule of the Isala Clinics to the optimal OR schedules created by our ILP model. In the total costs, the priorities of the stakeholders are included.

**Table 3:** Results scenario 4 and 5

Total penalty costs	Initial OR schedule		Realized OR schedule	
	Original	Optimal	Original	Optimal
Cancellation	0.00	0.00	66.67	22.22
Patient	0.00	0.00	40.91	36.50
Ward	0.00	0.00	34.40	28.56
Holding	0.00	0.00	0.00	0.00
OR assistants	0.00	0.68	6.43	4.20
Recovery	4.93	0.74	8.85	3.11
Radiology	1.63	0.56	2.41	1.07
Pathology	0.64	0.15	0.70	0.51
Logistics	0.00	0.00	9.26	5.93
Total costs	3.00	1.03	87.03	35.82

Table 3 shows that in Scenario 4, the initial OR schedule, the total penalty costs for the recovery and radiology department decreases. However, this can only be achieved by scheduling some surgeries in overtime. This follows from the slight increase of the total penalty costs for the OR assistants. For Scenario 5, the realization, the results show that the objective function value is reduced with approximately 60%. The major decrease is caused by the reduction of the number of cancellations. Also, the total penalty costs for the recovery department decreases significantly. In practice, the penalty costs for the realized OR schedule will lay somewhere between 35.82 en 87.03 when the DSS, discussed in the next section, is used.

Concluding, our model can potentially improve the initial and realized OR schedule significantly.

## Decision support system

To make our method applicable in practice, we have developed a DSS which can be used by the OR manager. We incorporated the two decision rules that are derived from the results in Section 3.2. The first decision rule is that the order of surgeries must be maintained, but that a surgery can be shifted in time. In addition, the second decision rule states that it is allowed to schedule a break of at most one hour between two surgeries. This may help to decrease the perceived workload of several stakeholders and may be necessary to fulfil the resource constraints.

**Fig. 9:**
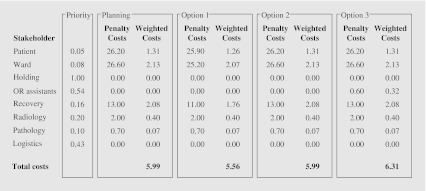
Decision support system - penalty costs

**Fig. 10:**
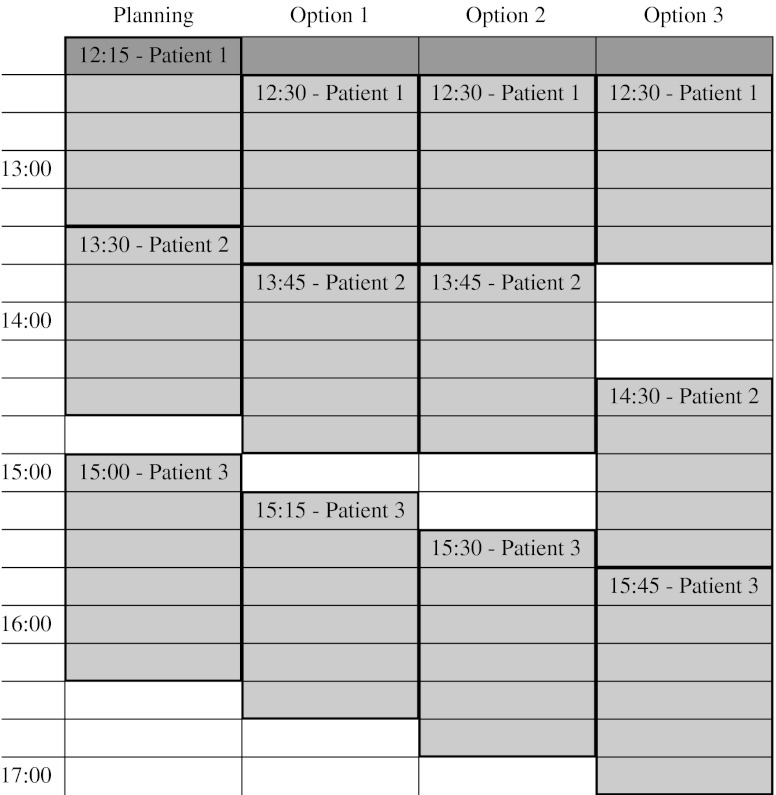
Decision support system - Gantt chart

During the day, the OR schedule must be adjusted, because of arriving emergency surgeries and elective surgeries that take shorter or longer than expected. The user can indicate for which OR the schedule should be adjusted. Because we have to consider all preferences and restrictions with respect to the other ORS, adjusting the schedule of the chosen OR is not straightforward. However, since we only have small instances, we evaluate, by means of complete enumeration, all possible solutions for this OR with respect to the two decisions rules. This means that between each two surgeries a break is scheduled with a duration that varies between 0 and 4 time periods. When *n* surgeries have to be rescheduled, this results in 5^*n* − 1^ possible solutions. In most cases *n* will be smaller than 5, which gives approximately 100 possible solutions. After all possible solutions are evaluated, the DSS presents the three best options to the user. Only feasible solutions with respect to the constraints described in Section 2 are considered. A screen-shot of the DSS is shown in Fig. 9. The first column of the screen-shot gives the specified priorities of all stakeholders. These values can be changed to create, for example, a patient centred OR schedule. The next column shows the penalty costs and weighted costs of the current OR schedule. The last three columns show the three best OR schedules with their penalty costs and weighted costs from which the user can choose. In addition, the DSS provides a Gantt chart of the current OR schedule and the three best feasible OR schedules (see Fig. 10).

The DSS gives insight in how other departments are influenced by a change in the OR schedule by denoting the penalty costs and weighted costs incurred for each stakeholder. This can convince surgeons that it can be useful to schedule a break between two surgeries. In addition, the DSS can determine whether the initial OR schedule is feasible or not by checking all constraints given in Section 2. The system denotes for each constraint how many times it is violated in the proposed OR schedule. It furthermore can be used to adjust the schedule such that it is feasible. Also, the penalty costs for the proposed OR schedule are calculated which gives an indication of how good the schedule is. The last advantage of the DSS is that the realized OR schedule can be evaluated. This way, the OR manager can learn from his decisions made in the past.

## Simulation study DSS

To determine the effect of using the DSS in practice, we tested the developed DSS on the instance given in Section 3 by means of a simulation study. In this study, we simulate the use of the DSS by rescheduling an OR immediately when it is disturbed. We choose to evaluate this rescheduling strategy, because we believe that this strategy will provide the best results.

At the beginning of each of the days in the considered instance, only the information that at that time is also known in practice is given. More specific, only the initial OR schedule is known, which is defined by the assignment of the elective surgeries to an OR and the initially planned start times *P*_*i*_ of the elective surgeries together which their expected duration *E*_*i*_. This means that the emergency surgeries are not known in advance in contrast to the considered Scenario 5.

For each point in time during the simulation, we insert the arrived emergency surgeries to the OR schedule and we update the surgery duration for each of the ongoing surgeries. For each arrived emergency surgery, we set the planned start time equal to their time of arrival and set their duration equal to the expected surgery duration. In addition, each emergency surgery has to be performed in the same OR as it was performed in the realized OR schedule of the Isala Clinics.

For the ongoing surgeries, we update the duration according to the following rules. When the realized duration of a surgery is less than the expected duration, we set the duration equal to the expected duration until the surgery is finished. At the moment in time the surgery is finished, we set the duration equal to the realized duration. When the realized duration of a surgery is larger than the expected duration, we set the duration equal to the maximum of the expected duration and the already executed duration thus far. When the already executed duration equals the realized duration, the surgery is finished and thus, the duration is set to the realized duration. In the next step of our simulation, we determine for each OR whether it becomes empty or whether an elective surgery which is scheduled to start at this point in time cannot be started. When an OR becomes empty, a new surgery may be started which could potentially improve the OR schedule for one or more of the stakeholders. In case an elective surgery which is scheduled to start cannot be started, we have the situation that an already started surgery assigned to the same OR takes longer than its expected duration. When one of these two cases occur, we reschedule this OR to decrease the penalty costs or to make the OR schedule feasible again. We reschedule this OR with respect to the fixed schedule in the other ORs as described in Section 4.

The results of the simulation study in Table 4 show that the DSS provides a much better solution than the original realized OR schedule of the Isala Clinics. However, as expected, the penalty costs for the DSS are higher than the penalty costs for the optimal realized OR schedule. When compared to the original realized OR schedule, the total penalty costs are reduced by approximately 50% for the DSS and by approximately 60% for the optimal realized OR schedule. In addition, the results show that the penalty costs for the patients and wards decrease when compared to the realized OR schedule of the Isala Clinics and the optimal realized OR schedule, however, the penalty costs for the OR assistants and the recovery, radiology and pathology departments increase. The penalty costs for the mentioned stakeholders increase because surgeries cannot be canceled or exchanged and thus, more surgeries have to be done in overtime. Concluding, the use of the DSS provides a better trade-off between the preferences of the involved stakeholders and by this reduces the incurred penalty costs significantly.

**Table 4:** Results simulation study DSS

Total penalty costs	Realized OR schedule		
	Original	Optimal	DSS
Cancellation	66.67	22.22	0.00
Patient	40.91	36.50	29.50
Ward	34.40	28.56	21.44
Holding	0.00	0.00	0.00
OR assistants	6.43	4.20	12.21
Recovery	8.85	3.11	14.70
Radiology	2.41	1.07	2.78
Pathology	0.70	0.51	1.14
Logistics	9.26	5.93	0.00
Total costs	87.03	35.82	45.13

## Conclusions

In this paper, we considered the problem of rescheduling surgeries on the day of execution. We formulated an ILP which determines the best adjusted OR schedule at a given point in time. The results show that patients, wards, and OR assistants have opposite interests compared to the recovery, radiology, pathology, and logistic department. Furthermore, the achieved results show that, without a few exceptions, the only used adjustments are (i) shifting surgeries, and (ii) scheduling breaks between two surgeries. These two decision rules are incorporated in a developed DSS. This system determines the best adjusted schedule for one OR with respect to the given constraints and gives insight in how the workload of stakeholders is influenced by adjusting the OR schedule throughout the day. The simulation study shows that by using this DSS, less surgeries are canceled and patients and wards are more satisfied, but also that the workload of several departments increases to compensate this.

A drawback of the developed DSS is that the decision rules may not be applicable when the priorities of the stakeholders change. A change in these priorities for the ILP can result in, for example, more exchanges or cancellations of surgeries. However, this is not expected in hospitals that have a similar group of stakeholders as the Isala Clinics, because these two adjustments are less preferred than shifting a surgery.

Another aspect of the DSS, which may be seen as a drawback, is that it only improves the OR schedule for one OR at a time. The idea for such a type of approach comes from the Shifting Bottleneck heuristic [1], where in each step the schedule of the bottleneck resource is optimized with respect to the schedule for the other resources. As for the Shifting Bottleneck procedure, our approach is only a heuristic approach and thus will not result in optimal solutions. However, the results of the simulation study show that the DSS performs relatively good when compared to the optimal OR schedule. In addition, the Isala Clinics do not prefer to reschedule multiple ORs at once, because then the process of optimization may be unclear to the user and the necessary changes to the OR-schedule at one point in time may be quite large resulting in a decrease of the acceptance of the achieved results.

Further research could focus on including the Central Sterile Supply Department (CSSD) into the model. This department prepares the instrument sets needed for a surgery. When a surgery is added to the OR schedule during the day, this may influence the workload on the CSSD. In addition, the CSSD may impose some extra constraints on the OR schedule.

There are several ways in which the developed DSS can be used, for example, reschedule an OR immediately when it is disturbed or reschedule all ORs at some moments in time. The last example also raises the question in what order the ORs should be rescheduled. Therefore, it would be interesting to investigate the best way to use the DSS.

## Figures and Tables

**Fig. 11:**
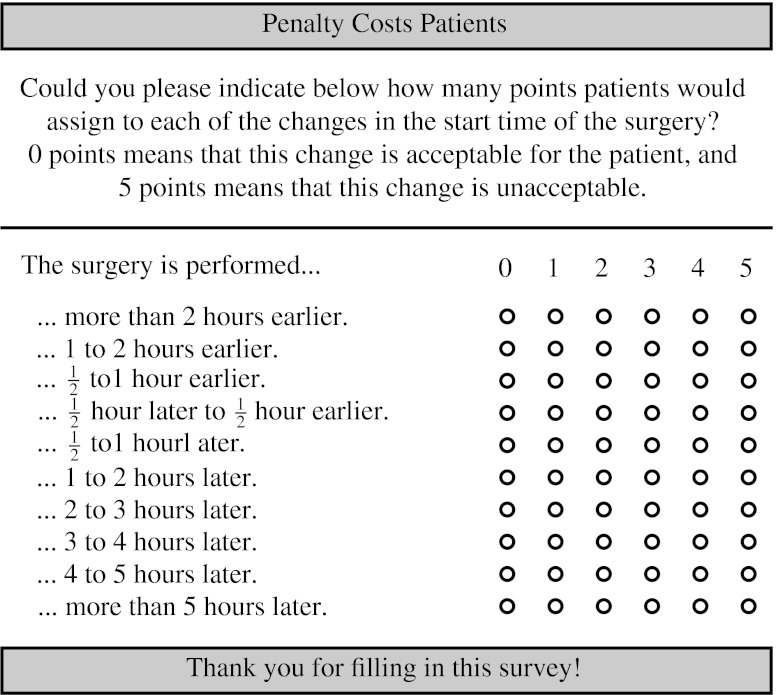
Survey patients preferences

**Fig. 12:**
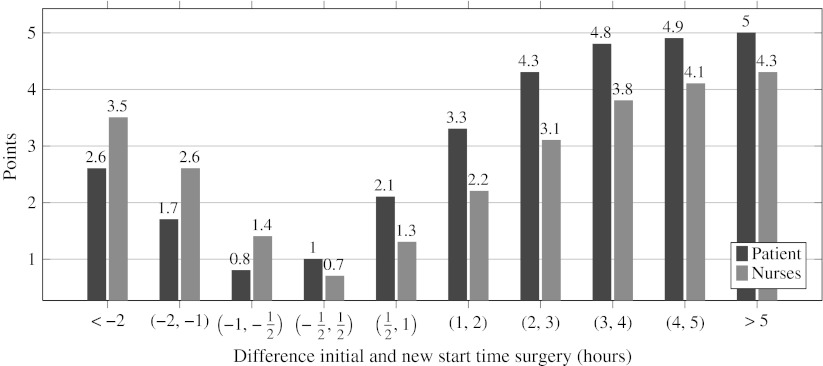
Results patients and nurses survey

**Fig. 13:**
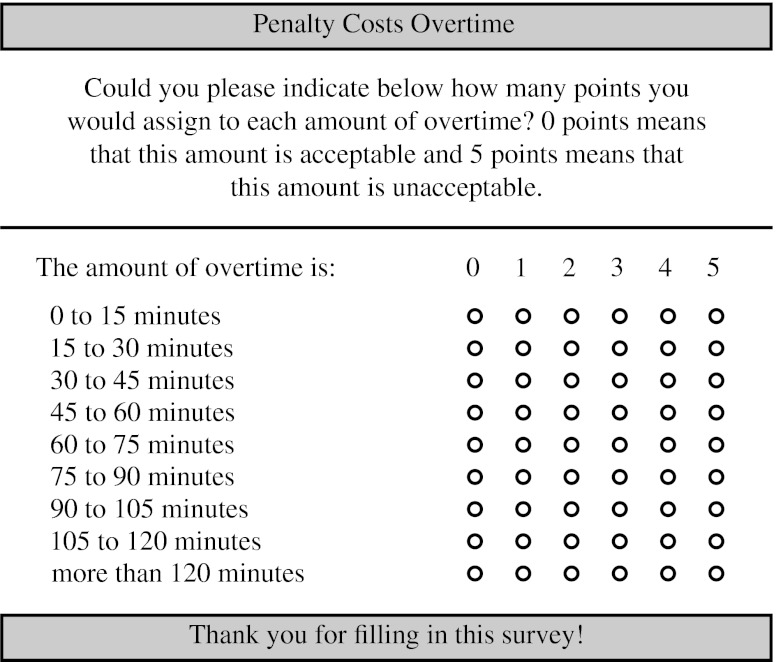
Survey OR assistants preferences

**Fig. 14:**
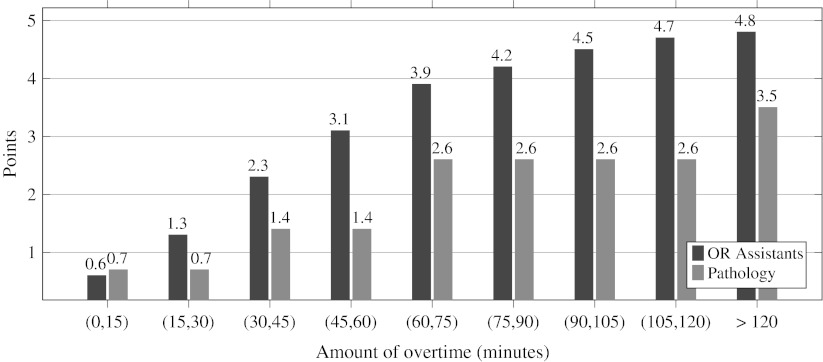
Results OR assistants and pathology survey

## Appendix

### A Survey

The preferences and restrictions of the stakeholders were determined by means of interviews and we conducted a survey to determine the penalty costs the stakeholders assign to changes in the OR schedule. In this appendix, we describe for each stakeholder the survey used and the resulting penalty costs.

#### A.1 Patient

By means of interviews with patients and nurses at the wards, we learned that patients do not prefer a change in the start time. Because it was not possible to ask the patients what penalty costs they would assign to different changes in the start time, we asked the nurses at the wards to fill in the survey. We received a completed survey from 29 nurses. The questions of the survey are depicted in Fig. 11 and the results in Fig. 12.

#### A.2 Ward

The interviews with the nurses at the wards also revealed that the nurses do not prefer a change in the start time of a surgery. Therefore, we asked the nurses to fill in the same survey as depicted in Fig. 11, however, this time, we asked them to indicate how many points they would assign to each of the changes in the start time of the surgery. We received a completed survey from 29 nurses for which the results are depicted in Fig. 12.

#### A.3 Holding department

Interviews with nurses at the holding department revealed that they prefer a levelled amount of patients at each point in time. The manager of the holding department indicated by assigning points which amount of patients is preferred by the department. The results can be found in Table 5.

**Table 5:** Results holding and recovery department survey

Number of patients	Points holding	Points recovery
0	0	0
1	0	0
2	0	1
3	0	3
4	1	5

#### A.4 OR assistants

By means of interviews with the OR assistants, we learned that OR assistants prefer a minimal amount of overtime. We asked the OR assistants to fill in a survey in which they could assign points to different amounts of overtime. The survey, depicted in Fig. 13, was filled in by 36 OR assistants. The results of this survey are shown in Fig. 14.

#### A.5 Recovery department

Interviews with nurses at the recovery department revealed that they prefer a levelled amount of patients at each point in time. The manager of the recovery department indicated by assigning points which amount of patients is preferred by the department. The results can be found in Table 5.

#### A.6 Radiology department

Interviews with radiology technicians revealed that they prefer to finish as soon as possible with their work at the OR such that they can assist at the radiology department. As measurement of deviating from this preference, we take the percentage of time radiology technicians are present but not working at the OR. The bigger this percentage, the more points are assigned. The assigned points per deviation are shown in Table 6.

**Table 6:** Results radiology department

Percentage	Points
0–10%	0
10–25%	1
25–37.5%	2
37.5–50%	3
50–60%	4
>60%	5

#### A.7 Pathology department

By means of interviews with the pathology department, we learned that the employees prefer a minimal amount of overtime. We asked the manager of the pathology department to assign points to different amounts of overtime, where 0 point means that the amount of overtime is acceptable and 5 points means that the amount of overtime is unacceptable. The results are shown in Fig. 14.

#### A.8 Logistic department

From an interview with the logistic department we learned that logistic employees do not want surgeries to be swapped. The logistic department assigned 5 points, the maximum amount, to swapping two surgeries.
